# *Saccharomycopsis schoenii*: A Model Predatory Yeast Provides Insights into Evolution, Genomics and Biocontrol

**DOI:** 10.3390/jof12090651

**Published:** 2026-09-01

**Authors:** Divya Kriti, Marjan Barazandeh, Joseph Uche Ogbede, Guri Giaever, Corey Nislow

**Affiliations:** 1 Department of Biochemistry and Molecular Biology, University of British Columbia, 2350 Health Sciences Mall, Vancouver, BC V6T 1Z3, Canada; dkriti@student.ubc.ca; 2 Department of Pharmaceutical Sciences, University of British Columbia, 2405 Wesbrook Mall, Vancouver, BC V6T 1Z3, Canada; marjan.barazandeh@ubc.ca (M.B.); ucheogbede@gmail.com (J.U.O.); g.giaever@ubc.ca (G.G.)

**Keywords:** *Saccharomycopsis schoenii*, mycoparasitism, necrotrophy, biocontrol, secreted aspartic proteases, comparative genomics, CUG-Ser2 clade

## Abstract

Fungal mycoparasitism, where one fungus preys on another, offers a sustainable, eco-friendly alternative to synthetic chemical fungicides in agriculture and conventional antifungal therapies to manage clinical pathogens. The budding yeast *Saccharomycopsis schoenii* has emerged as a compelling model for contact-dependent, necrotrophic predation. Belonging to the CUG-Ser2 clade, its obligate predatory lifestyle is characterized by extensive genomic restructuring, including the loss of the sulfate assimilation pathway, which couples methionine starvation to a predatory behavioral switch. In this review, we highlight recent comparative genomics and functional studies as they relate to *S. schoenii* predation. For example, *S. schoenii* uses rewired mitogen-activated protein kinase (MAPK) signaling, actin-based penetration pegs and a large tandem expansion of secreted aspartic proteases (SAPs) to breach prey cell walls. Analysis of its genome reveals structural adaptations, including AT-rich regional centromeres and dispersed mating-type (MAT) loci. The more we learn about *S. schoenii*’s contact-dependent attack, the greater the opportunities to deploy *S. schoenii* as a safe, mycotoxin-free biocontrol agent against both multi-drug-resistant *Candida auris* and post-harvest *Penicillium* molds.

## 1. Introduction

The rising incidence of antifungal resistance in agricultural and clinical settings requires the development of alternative pathogen management strategies [1,2]. Traditional chemical fungicides and clinically used antifungal drugs are losing efficacy due to the emergence of multi-drug resistant pathogens, such as *Candida auris* and post-harvest *Penicillium* molds [1,3,4]. The continuous use of such fungicides also raises environmental, ecological, and toxicological concerns [2,5]. Biological control agents represent a sustainable alternative [6]. Filamentous fungi like *Trichoderma* species are well-documented biocontrol agents, but they typically rely on the secretion of secondary metabolites and mycotoxins, which can pose cytotoxic, off-target risks [7,8]. Predatory yeasts offer a different approach because they rely on contact-dependent mechanical and enzymatic methods to kill fungal prey. This avoids the emergence of traditional drug resistance without requiring broad-spectrum chemical toxins [3,9].

Despite their utility, predatory yeasts are quite underrepresented in biocontrol research compared to their filamentous counterparts [3,10]. Significant knowledge gaps remain regarding the molecular and evolutionary mechanisms governing yeast mycoparasitism. Historically, highly fragmented draft genomes have hindered the analysis of the structural adaptations and gene family expansions that shape necrotrophic behavior in non-model organisms [9,11,12]. The evolutionary transition from saprotrophy to an obligate predatory lifestyle and how this correlates with distinctive traits like alternative genetic codes and metabolic dependencies, such as sulfate auxotrophy, requires further clarification [9,13,14].

This review addresses these gaps by consolidating recent genomic, transcriptomic, and functional data on the emerging model predatory yeast *Saccharomycopsis schoenii* [3,9,15,16]. The primary purpose of this work is to explain the evolutionary and molecular basis of contact-dependent mycoparasitism in yeasts and evaluate their feasibility as safe, effective biocontrol agents.

The main body of this review is divided into four sections. First, we outline the general context of fungal predation, comparing biotrophic and necrotrophic attack strategies. Second, we detail the evolutionary origins of predatory yeasts, focusing on clade-specific codon reassignment and how metabolic starvation acts as a trigger for the predatory lifecycle. Third, we examine the structural genomic adaptations and expanded hydrolytic arsenals that enable *S. schoenii* to physically breach prey cell walls. Finally, we assess the practical applications of *S. schoenii* in controlling multi-drug resistant and agricultural pathogens, concluding with an analysis of its biosafety profile.

## 2. Fungal Predation: From General Context to Biotrophic and Necrotrophic Strategies

### 2.1. The Broader Context of Fungal Predation

Accounting for an estimated 2.2 to 3.8 million species, fungi are ubiquitous and thrive in many different ecological environments [17]. Traditionally, they are thought of as saprophytic decomposers that recycle complex organic matter, or as mutualistic symbionts that form beneficial mycorrhizal partnerships with plants [18,19]. A less explored but significant aspect of mycology is fungal mycoparasitism, where one fungus actively preys on another for food or habitat [20]. These antagonistic interactions drive microbial population dynamics and nutrient cycling within ecosystems, for example, by breaking down fungal cell walls and releasing the nutrients inside to make these resources available to other organisms [21,22]. They also shape fungal communities, acting as natural biocontrol agents by suppressing the unchecked proliferation of pathogenic fungal populations through highly regulated mechanisms of hyperparasitism, nutrient competition, and antibiosis [23,24]. Mycoparasites therefore represent a valuable, relatively untapped reservoir of potential biological control agents. By way of example, *Trichoderma* species have been used against soil-borne pathogens and *Ampelomyces quisqualis* against powdery mildews, offering sustainable and eco-friendly alternatives to conventional chemical fungicides [6].

**Figure 1:**
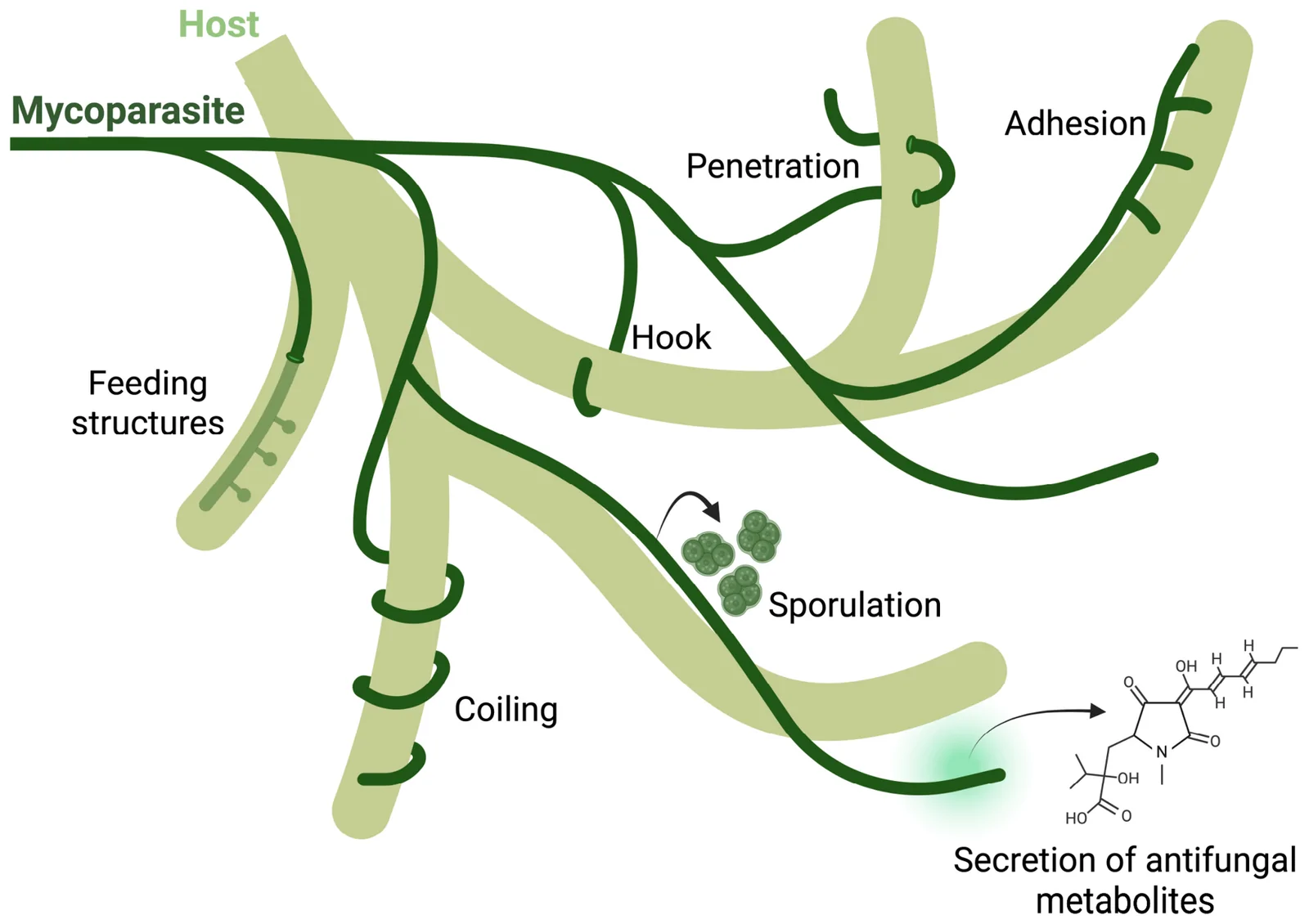
Overview of mycoparasitic interactions. Schematic of a mycoparasitic fungus (dark green) attacking a filamentous fungal prey (light green). Mycoparasites use multiple strategies, including siphoning resources from their prey: adhesion to the host hypha, coiling around the host, formation of hooks and penetration pegs to breach the host wall, development of internal feeding structures, sporulation on or near the host, and secretion of antifungal metabolites (e.g., harzianic acid). (Created in BioRender. Kriti, D. (2026) https://BioRender.com/1ohs6eo).

Mycoparasitic fungi employ various strategies to detect, attack, and siphon resources from their prey [10,20]. These mechanisms, which likely reflect a co-evolved parasitic relationship, include adhesion to the host hypha, coiling around the host, forming hooks and penetration pegs to breach the host wall, developing internal feeding structures, sporulating on or near the host, and secreting antifungal metabolites [10,25,26] (Figure 1).

### 2.2. Biotrophic vs. Necrotrophic Mycoparasitism

Functionally, mycoparasitism is classified as biotrophism or necrotrophism. Biotrophic mycoparasites form specialized structures to establish enduring attachments with their hosts. They gradually extract nutrients using structures such as haustoria or intracellular hyphae, ensuring that the host cell remains viable and metabolically active to serve as a continuous nutrient source [27]. At the molecular level, biotrophic predation is characterized by localized secretion of cell wall-degrading enzymes (CWDEs), which allows for targeted penetration without activating massive host damage response [28]. To evade detection, biotrophs use small secreted proteins (SSPs) that actively suppress host immune signaling and halt the hypersensitive response [29]. For example, in well-characterized plant-pathogen systems that serve as the conceptual model for fungal mycoparasitism, the Pep1 effector from corn smut fungus, *Ustilago maydis* blocks the host’s oxidative burst to maintain cellular viability and ensure a continuous nutrient source [30]. Emerging evidence suggests mycoparasites deploy similar effector-driven strategies [20,31].

Necrotrophic mycoparasites, in contrast, act as aggressive predators. They rapidly kill their prey before scavenging and consuming cellular material using a combination of directed mechanical pressure, potent cell wall-degrading enzymes, and reactive oxygen species (ROS) [20,25]. Necrotrophs typically exhibit a broader host range than biotrophs [10]. While their attack still requires them to detect the presence of the host (typically through surface G-protein coupled receptors that sense host-derived signaling oligomers [7,32]), their primary predation strategy relies on the rapid enzymatic degradation of fungal structural components, such as chitin and β-glucan [20,33]. This approach bypasses the gene-for-gene effector compatibility required to maintain biotrophic interactions [28,29]. Necrotrophs typically form distinct infection structures. Rather than forming nutrient-siphoning haustoria, necrotrophs: (1) use hyphal coiling around their prey, (2) develop appressoria-like infection pads, or (3) form penetration pegs to physically breach their prey [3,26,34].

To carry out predation, necrotrophs rely on secreted hydrolytic enzymes where chitinases break down chitin in fungal cell walls, while glucanases target structural polysaccharides, and proteases degrade host proteins [20,31]. These enzymes are sometimes accompanied by an expanded set of SSPs or effector proteins to help breach host defenses. LysM effectors such as *ECP6*, secreted by a fungal plant pathogen, bind chitin fragments and suppress host immune recognition [35], while necrosis and ethylene-inducing proteins (NLPs) directly trigger host cell death [36]. While in vitro assays can achieve near-total prey eradication, ecological success rates vary based on environmental variables like humidity, nutrient availability, presence of resilient host resting structures, etc. The genomes of necrotrophs frequently display evidence of gene family expansion, along with indications of horizontal gene transfer, and other regulatory adaptations that are directly linked to their predatory lifestyle [15,33,37]. For instance, glycoside hydrolase and peptidase families are expanded in *Trichoderma* species, consistent with their capacity to degrade host cell walls [33]. Similarly, horizontally acquired secondary metabolite clusters and transporter genes, such as ABC and MFS transporters, are frequently expanded, suggesting an adaptive role in chemical defense and metabolite exchange during host–parasite interactions [10,33,38]. Mechanistic studies show that in necrotrophic mycoparasites environmental cues are processed via surface recognition systems like G-protein coupled receptors and subsequently routed through MAPK signaling frameworks. This allows the necrotroph to detect host-derived signals and initiate colonization [7]. A classic example is the *TMK1* MAP kinase directing host sensing and attack in *Trichoderma atroviride* [39]. Table 1 describes the features and strategies of biotrophs and necrotrophs in detail.

## 3. Evolutionary Origins of Predatory Yeasts

### 3.1. Ecological Diversity Within the Saccharomycopsis Genus

*Saccharomycopsis* is the sole genus within the family Saccharomycopsidaceae, comprising 19–24 recognized species that span a wide variety of metabolic and ecological niches [54,55,56]. The *Saccharomycopsis* genus is a striking example of evolutionary and functional diversification within budding yeasts. Species are found across a wide range of ecological contexts, from decaying plant material to fermenting foods, reflecting adaptations to distinct nutrient environments [57]. For example, *Saccharomycopsis fibuligera* and *Saccharomycopsis capsularis* are non-predatory, amylolytic yeasts known for their ability to degrade starch and proteins [13,58,59]. *S. fibuligera* is utilized extensively in traditional Asian rice wine and beer fermentations because it produces large amounts of amylases and glucoamylases that convert raw starch into fermentable sugars [58,60,61], while *S. capsularis* is primarily recognized as a pellicle-forming spoilage agent that degrades fermented products like hot pepper paste [62].

In contrast, the organic sulfur auxotroph *Saccharomycopsis schoenii* relies on predation as a survival strategy [13]. Because it targets a broad range of fungal prey, it is a promising candidate for biocontrol applications across medicine, agriculture, and winemaking [3,4,16]. Other species within the genus are also adapted to specialized, nutrient-scarce environments that favor antagonism. *Saccharomycopsis fodiens* was originally isolated from flower-associated nitidulid beetles [63], while *Saccharomycopsis javanensis* is frequently found in oak tree fluxes and insect-associated microbiomes [13]. Despite this diversity, the genera share characteristic traits, including unusual nutritional requirements, deviations from the canonical genetic code, and, in certain species, a predatory lifestyle [13,14].

### 3.2. The CUG-Ser2 Clade and Codon Reassignment

The *Saccharomycopsis* genus belongs to the order Ascoideales, which includes only two genera: *Saccharomycopsis* and *Ascoidea* [14]. A hallmark of this order is its non-standard genetic code. The CUG codon, which encodes leucine in standard eukaryotes, is translated as serine. Consequently, this lineage is referred to as the CUG-Ser2 clade [9,14,64]. This leucine to serine change was achieved through the duplication of an ancestral *tRNA-Ser* gene, followed by a mutation in its anticodon (to CAG), allowing it to recognize and translate CUG codons as serine [14]. Genomic studies reveal that, while all protein translation in *Saccharomycopsis* uses the tRNA-Ser(CAG), many species within the genus also retain an ancestral, non-functional *tRNA-Leu(CAG)* gene, the exact non-translational function of which remains a subject of active investigation [9,14]. This codon reassignment is shared with the order Serinales, which includes the well-known CUG-Ser1 clade (historically referred to as the CTG-clade) pathogens such as *Candida albicans*, *Candida tropicalis*, and the multi-drug resistant *Candida auris* [14,65,66].

Translation of CUG as serine fundamentally alters the proteome. Since serine is a small, polar amino acid and leucine is a bulky, hydrophobic one, this substitution can alter protein folding, structural stability, and enzymatic active sites [67]. This code alteration also acts as a robust genetic barrier to horizontal gene transfer [14]. In other words, if a standard-code yeast transfers a functional gene containing multiple CUG codons into a *Saccharomycopsis* species, the translation machinery of the predator will misincorporate serine at every leucine position, potentially resulting in non-functional, misfolded, or toxic protein products [14,67]. While this codon reassignment acts as a robust genetic barrier to horizontal gene transfer [14], it may have initially provided an evolutionary edge. Evidence in *Candida* species suggests that CUG ambiguity and the resulting proteome instability induced a general stress response and phenotypic diversification, ultimately aiding adaptation to new ecological niches [68,69]. This forced genetic isolation likely facilitated the genus’s distinct evolutionary trajectory, allowing specialized traits, such as the tandem expansions of secreted aspartic proteases and the complex spatial reorganization of the mating-type loci into macro-palindromes, to diverge and become established within the population [15]. This mistranslation barrier also restricts mobile genetic elements [14,15]. Consequently, the unique genetic code of *S. schoenii* is both a taxonomic marker and a catalyst of its highly specialized, necrotrophic lifestyle, which is driven by a complex interplay of this genetic isolation, the targeted expansion of hydrolytic gene families, rewired regulatory mechanisms, and specific environmental starvation signals [9,15].

### 3.3. Sulfate Auxotrophy Drives Predation

Beyond codon reassignment, the *Saccharomycopsis* genus shows significant metabolic streamlining, most notably in its inability to assimilate inorganic sulfate. Comparative genomic analyses confirm the genus has lost multiple critical biosynthetic genes required for this pathway, rendering it entirely dependent on environmental or prey-derived organosulfur compounds, such as methionine [3,13,16]. This liability is offset by predation, where *Saccharomycopsis* species extract sulfur directly from the cytoplasm of their lysed prey [13]. To efficiently scavenge these released nutrients, the *S. schoenii* genome has evolved an expanded repertoire of sulfur permeases and transporters [9].

This loss of the sulfate assimilation pathway highlights a broader transition from saprotroph to predator, driven by genomic plasticity. As these yeasts evolve to rely on complex biomolecules from their environment or their prey, maintaining energy-intensive de novo biosynthetic pathways becomes a liability [70,71]. Consequently, speciation within *Saccharomycopsis* is frequently characterized by the rapid contraction of canonical metabolic pathways [9,14]. Beyond sulfur, this decay extends into carbohydrate metabolism. For example, the *GAL* gene cluster, which controls galactose metabolism in model yeasts, is absent or heavily degraded [16,72]. This gene shedding suggests a specialized dietary shift, where the need to metabolize diverse sugars was lost in favor of scavenging intracellular prey nutrients. Similar genomic plasticity is observed in nitrogen metabolism, marked by variations in the retention of genes governing the assimilation of non-preferred nitrogen sources, such as specialized amino acids and complex nitrogenous compounds [13,73]. In summary, these predatory species tend to develop specialized nutrient import pathways after losing the genes needed to synthesize their own nutrients and have instead evolved additional transporters to absorb amino acids and simple carbohydrates from their prey’s cytoplasm [9,15,73].

### 3.4. Predatory Lifecycle of S. schoenii

**Figure 2:**
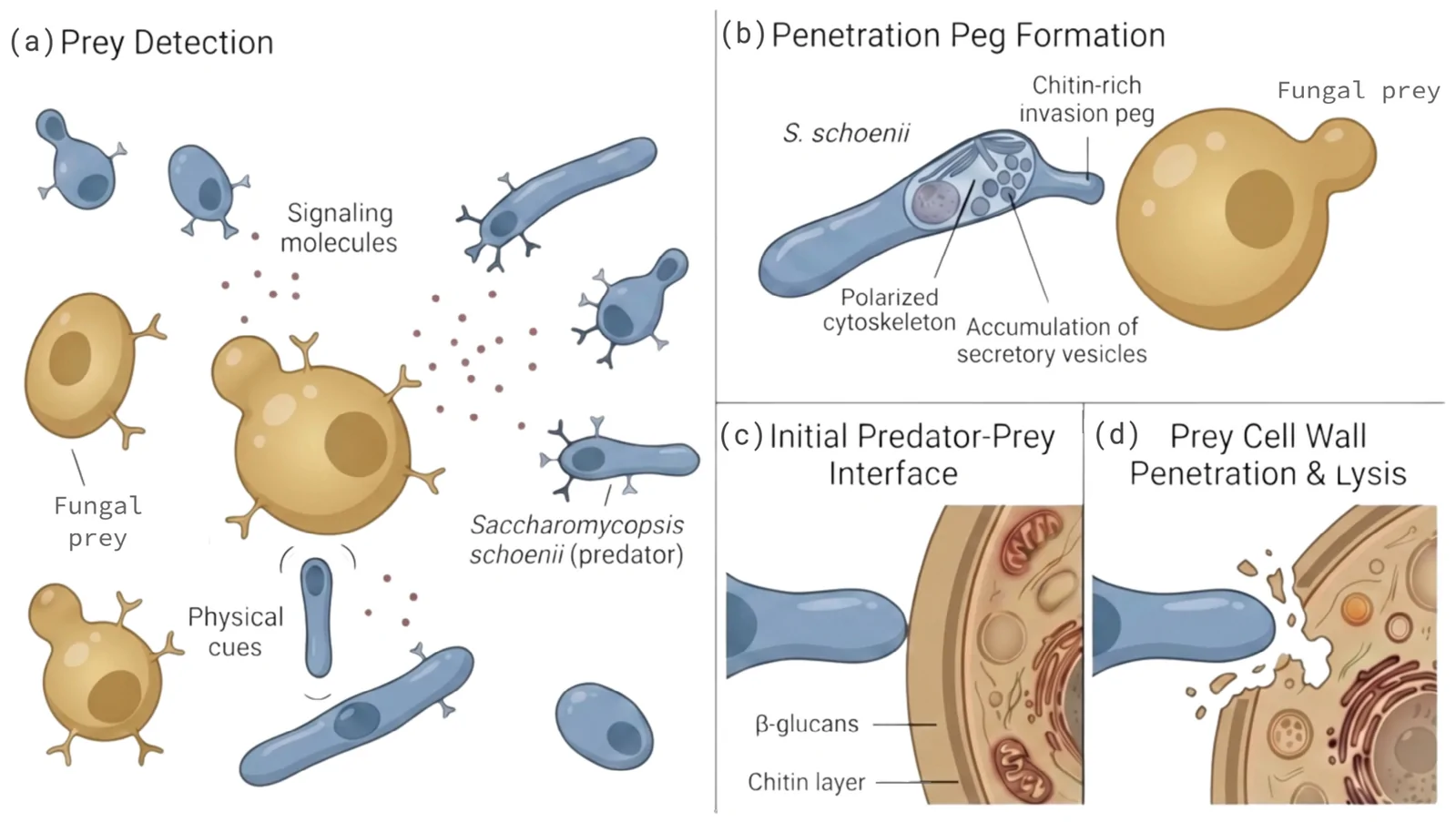
Predation by *S. schoenii*. The figure shows stages of contact-dependent mycoparasitism by *S. schoenii* against a fungal prey cell. (**a**) Prey detection: the predator senses nearby cells via chemical and physical cues and orients growth toward the target. (**b**) Penetration peg formation: a chitin-rich, polarized protrusion develops at the predator apex and advances toward the prey. (**c**) Contact zone: the predator establishes a focused interaction with the prey. (**d**) Cell wall breach and lysis: the peg locally disintegrates the prey cell wall, and *S. schoenii* begins nutrient uptake. (Created in BioRender. Kriti, D. (2026) https://BioRender.com/r97qn32).

*S. schoenii* has emerged as a robust experimental system for studying contact-dependent mycoparasitism. Recently, there has been an increase in interest in its ability to attack a wide range of fungi, including *Candida* species, some of which are notorious for their multi-drug resistance [3,9]. It has been shown to kill phytopathogenic molds such as *Penicillium digitatum*, *Penicillium italicum*, and *Penicillium expansum* [4]. The mechanism is direct and efficient: upon recognition and close contact with prey, *S. schoenii* forms penetration pegs that breach the prey’s cell wall, leading to rapid cell death of the prey and subsequent consumption of released nutrients [3,16] (Figure 2).

Recent studies have addressed the molecular basis of *S. schoenii* predation. Transcriptomic and proteomic profiling shows that predation induces the upregulation of secreted aspartic proteases, carbohydrate-active enzymes (CAZymes), and diverse redox-buffering and stress-responsive proteins [9,15]. It is a coordinated process, influenced by nutrient availability and environmental conditions. Specifically, environmental cues such as organic sulfur limitation (especially methionine) trigger its transition to a predatory state [9,13]. Rij et al. (2024) showed that rewiring of conserved MAP kinase signaling pathways is required for penetration peg formation and successful predation [16].

*S. schoenii* predation is not a passive trait, but rather represents a coordinated response influenced by nutrient availability and environmental conditions [9]. For a successful necrotrophic attack, the predator relies on three important physiological and molecular steps:

• Starvation triggers predation in *S. schoenii*: *S. schoenii* cannot synthesize essential sulfur-containing amino acids, such as methionine, de novo, and therefore relies on external organic-sulfur compounds [9,13]. This metabolic limitation makes predation advantageous under sulfur starvation [13]. When environmental sulfur is depleted, *S. schoenii* extracts pre-synthesized methionine and other nutrients from nearby prey fungi [9,16]. Current data indicates this behavior is strictly fungivorous, likely due to the predatory interaction relying on recognizing and enzymatically breaching highly specific fungal cell wall components (such as β-glucans and chitin) [9,13]. To facilitate this scavenging, its genome retains multiple transporters like *MUP3* (low-affinity methionine permease), *YCT1* (cystine transporter), and *SEO1* (high-affinity dipeptide transmembrane transporter) [9]. When *S. schoenii* ruptures a host fungal cell, it rapidly absorbs the intracellular metabolites (methionine and cysteine) released from the lysed fungal prey. In other words, the absence of sulfate assimilation genes couples starvation to a predatory switch;
• Prey detection and rewiring of MAPK signaling in *S. schoenii*: A necrotrophic attack requires a timely molecular response. Recent functional studies show how *S. schoenii* predates its fungal prey. Before using its hydrolytic capacity, the predator relies strictly on environmental sensing and physical contact to trigger the predatory offense [3,9]. It actively tracks and orients toward its host via a sophisticated signal transduction network [16]. At the core of this network is an evolutionary adaptation: the predator has co-opted and rewired the highly conserved MAPK pathways [16]. In standard non-predatory yeasts, these cascades govern basic pheromone responses and filamentous growth, but in *S. schoenii* they have been reprogrammed to drive necrotrophy [16]. Functional analyses reveal that specific MAPK nodes and their downstream transcription factors now serve as the master regulators of pathogenicity. For example, both the MAPK Kil1 and the transcription factor Ste12 are essential for predatory morphogenesis [16]. Deleting the genes that encode these proteins strips the yeast of its ability to initiate an attack [16];
• Morphogenesis and Penetration Peg Formation: Following successful prey recognition and MAPK signaling, the predator executes a rapid morphogenetic shift. *S. schoenii* polarizes its actin cytoskeleton to construct a specialized, chitin-rich invasion structure known as a penetration peg [3,16]. This peg exerts focused mechanical pressure against the prey’s outer defenses. Because mechanical force alone is insufficient to breach the highly cross-linked glucan matrices of a target fungal cell wall, this physical intrusion is usually aided by a secretory burst [9]. The predator rapidly shuttles vesicles to the interaction zone, flooding the host interface with a broad-spectrum mix of hydrolytic enzymes, such as the secreted aspartic proteases and CAZymes [9,15]. Transcriptomic profiling reveals that this enzymatic burst is characterized by upregulation of SAP and CAZyme families upon engaging with prey cells. To survive the ROS bursts generated during this cellular breach, the predator uses redox management systems (such as superoxide dismutases and alternative oxidases) to protect its own cellular machinery while predating [9]. Following host cell lysis, *S. schoenii* rapidly absorbs the released intracellular metabolites through its expanded permeases [9]. This influx of essential organosulfur nutrients restores metabolic homeostasis, which then represses the starvation signals that initially triggered the predatory switch [9]. Once the localized prey resources and environmental sulfur are exhausted again, the yeast resumes its predatory lifecycle [13,16].

## 4. Genomics

For non-model organisms like *S. schoenii*, a contiguous, accurately annotated reference genome is important for interpreting complex structural variations and gene family expansions/contractions [11,15]. For example, a high-quality reference for *C. rosea*, uncovered expanded secondary-metabolite gene clusters and large *ABC*/*MFS* transporter repertoires, which guided functional tests showing that specific polyketide synthase genes shape antagonism against *B. cinerea* and *Fusarium graminearum* [38,74,75]. This shows that a high-quality genome assembly can help in mapping gene function, genomic architecture, gene family expansions, and synteny, which in turn aids functional annotation [76,77].

### 4.1. High-Resolution Genome Assembly

Historically, fungal genomics relied on short-read sequencing technologies. While these platforms provide deep sequence coverage at a relatively low cost, they do not resolve complex genomic topographies [12]. Short-read assemblies frequently collapse repetitive regions, expansive centromeres, and large transposable elements, resulting in highly fragmented reference genomes [12,78].

The first published genome of *S. schoenii*, sequenced in 2019, illustrates this bottleneck [9]. This draft genome was fragmented and lacked structural information, such as chromosomal linkages and repetitive elements. To overcome these structural limitations, recent investigations into *S. schoenii* used a hybrid genomic approach [15]. The combination of long-read sequencing (Pacific Biosciences High-Fidelity reads) with high-throughput chromosome conformation capture (Hi-C) scaffolding preserved the three-dimensional spatial arrangement of the intact nucleus and resolved the 14.3 megabase (Mb) *S. schoenii* genome into seven chromosome-scale scaffolds: six nuclear chromosomes and one circular mitochondrial chromosome [15]. High-quality genomes also enable high-resolution comparative genomics.

### 4.2. Comparative Genomics and Gene Family Evolution

**Figure 3:**
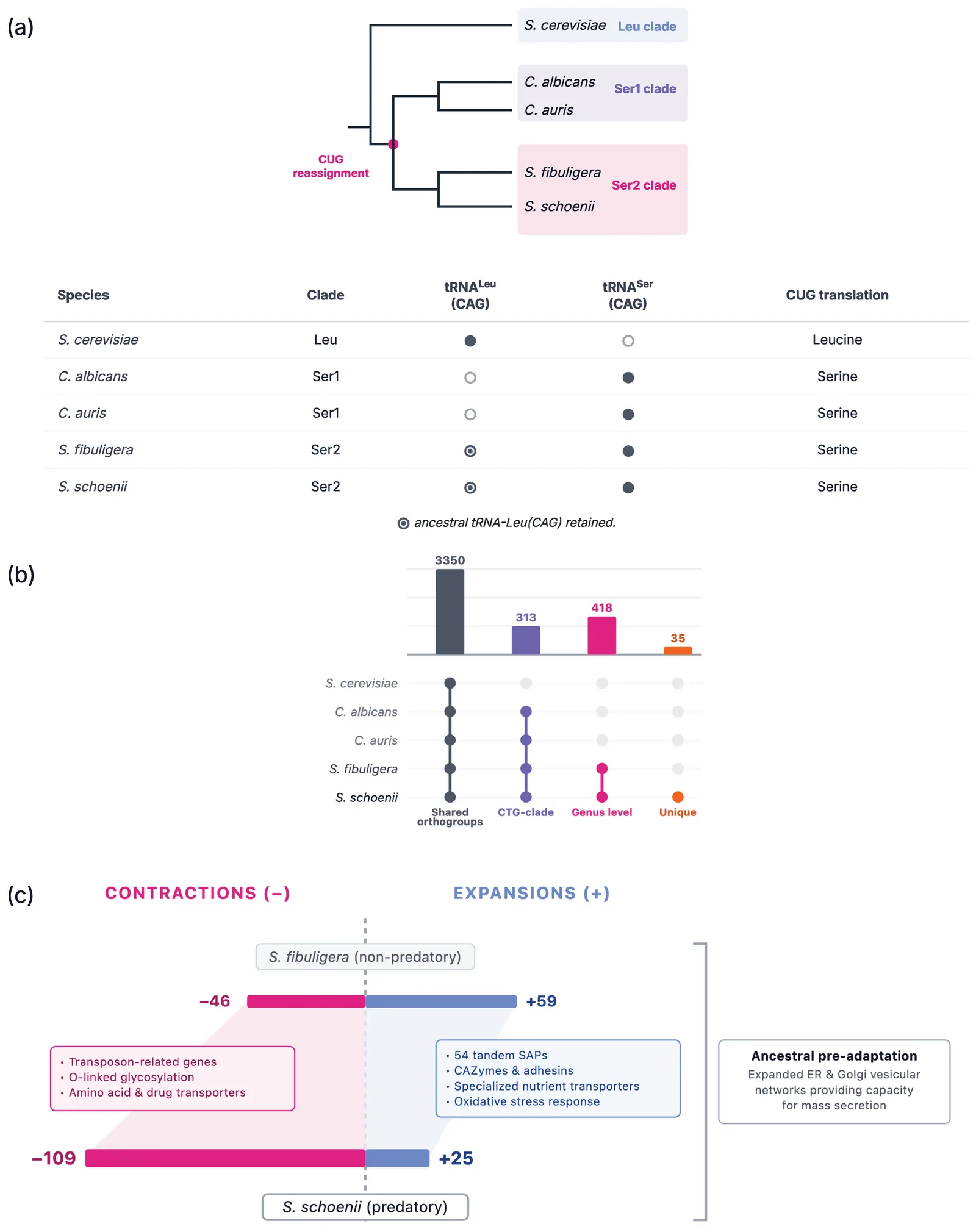
Evolutionary mapping and genomic adaptations of *S. schoenii*. (**a**) Cladogram of budding yeast clades and accompanying table show tRNA retention (solid dots indicate tRNA retention, while open dots indicate their absense). The Ser2 (or CUG-Ser2) clade encompassing the *Saccharomycopsis* genus retains the ancestral *tRNA-Leu(CAG)* gene alongside the newly evolved *tRNA-Ser(CAG)*, which translates the CUG codon as serine [9,14]. (**b**) The upSet intersection matrix shows orthologous gene cluster distribution by comparing five diverse yeast genomes. The matrix highlights a foundation of 3350 core conserved clusters, alongside overlaps specific to the Ser1 (or CUG-Ser1) clade encompassing *Candida* species, genus-level adaptations shared with *S. fibuligera*, and 35 orthologous clusters unique to *S. schoenii* [15]. In the panel, solid dots connected by vertical lines denote the specific species that share the orthologous clusters in each intersection group, while light gray dots represent species excluded from that group. (**c**) Flow diagram compares gene family expansions (+) and contractions (−) between the non-predatory *S. fibuligera* and the predatory *S. schoenii*. Both share an ancestral pre-adaptation of expanded secretory networks. Relative to their common ancestor (grey dashed line), *S. fibuligera* shows 59 gene family expansions and 46 contractions, whereas *S. schoenii* is characterized by 25 specific expansions of its predatory arsenal and nutrient scavenging pathways and 109 targeted contractions that streamline its genome as well as the cell surface. Orthologous cluster counts, gene family counts and functional annotations referenced in the figure are derived from [3,9,14,15].

Genomic studies show that the enzymatic secretion systems, such as those used by *S. fibuligera* for carbohydrate degradation, provided a genomic framework (specifically, an expanded endoplasmic reticulum and Golgi vesicular trafficking network) that predatory species co-opted to secrete host-degrading hydrolytic enzymes [14,54,61] (Figure 3c).

This evolutionary trajectory (Figure 3a,b) is reflected in the finished *S. schoenii* genome. Orthology analysis comparing *S. schoenii* with its non-predatory relative *S. fibuligera*, the standard model yeast *Saccharomyces cerevisiae*, and the clinical pathogens *C. albicans* and *C. auris* identified 5685 orthologous gene clusters [15]. While the majority of these clusters represent highly conserved, core cellular machinery shared across all five species (3350 clusters), *S. schoenii* possesses a unique genetic signature (35 unique clusters). It also shares a distinct subset of orthologs exclusively with *S. fibuligera* (418 clusters), and 313 clusters with the CUG-Ser1 clade *Candida* pathogens that are absent in *S. cerevisiae* [15]. While functional annotations suggest these clusters are enriched for stress-response and nutrient-scavenging pathways, their relation to the necrotrophic shift remains correlative and requires functional validation [15].

Figure 3c shows that when directly compared to its non-predatory relative, the *S. schoenii* genome exhibits significant diversification. While *S. fibuligera* exhibits a relatively balanced genomic flux, *S. schoenii* is characterized by 25 major gene family expansions and 109 contractions, with the most prominent expansion being a tandemly duplicated array of 54 SAPs [15]. This hydrolytic toolkit is complemented by the expanded families of CAZymes and adhesins. Before hydrolytic enzymes can be effectively deployed, the predator must physically anchor itself to the prey. Expanded adhesin families mediate this initial, cell-to-cell contact, ensuring that secreted enzymes remain highly concentrated at the predator-prey interface rather than diffusing into the environment [3]. Once anchored, the targeted secretion of SAPs and CAZymes helps break the highly cross-linked glucan and glycoprotein networks in the prey cell walls [3,9,15]. Additional genome expansions in oxidative stress responses and specialized nutrient transporters further equip the predator for necrotrophy [15].

In contrast to these genome expansions, genomic contractions highlight traits made dispensable by a predatory lifestyle Figure 3c. The predator exhibits a significant reduction in transposon-related genes, indicating a shift toward genomic stabilization typical of obligate parasites [15,79]. Additionally, the reduction in O-linked protein glycosylation pathways suggests a structural change in the cell surface. For example, *S. schoenii* appears to lose complex carbohydrate layers used to evade host immunity and instead streamlines its exterior for immediate predatory engagement [15,80]. Finally, the loss of broad-spectrum amino acid and drug transporters points to an optimized metabolism designed to directly scavenge concentrated nutrients from lysed prey [9,15].

### 4.3. Expansion of Secreted Aspartic Proteases (SAPs)

The physical breach of a host cell during necrotrophic mycoparasitism requires mechanical force via penetration pegs as well as a coordinated secretion of specialized hydrolytic enzymes [20]. The *S. schoenii* chromosome-scale assembly reveals a massive tandem duplication of 54 SAPs [15]. These SAPs belong to the pepsin-like family of endopeptidases (MEROPS family A1) [81,82], characterized by a bilobed tertiary structure where conserved Asp32 and Asp215 residues coordinate a water-mediated nucleophilic attack in acidic environments [1,82,83]. To understand the evolutionary weight of this expansion, *S. schoenii* can be compared to the clinical pathogen *C. albicans*, which uses a smaller set of ten SAPs (Sap1-Sap10) as primary virulence factors [1]. It targets mammalian extracellular matrix components, using Sap1-Sap3 for mucosal barriers and Sap4-Sap6 for deep tissue penetration [1]. In contrast, the 54-gene SAP expansion in *S. schoenii* targets a fundamentally different structural barrier: the fungal cell wall. During predation, localized SAP secretion at the penetration peg tip helps puncture the dense outer matrix of host glycoproteins and mannoproteins. Subsequently, expanded arrays of CAZymes, primarily chitinases (glycoside hydrolase 18, GH18) and endo-glucanases (GH16), bind to and degrade the underlying rigid 1,3-glucan, 1,6-glucan, and chitin matrix, leading to loss of host cell wall integrity and cytolysis [2,9,20].

### 4.4. Genomic Organization and MAT Locus Architecture

The *S. schoenii* genome features expansive, AT-rich regional centromeres that act as Cse4-nucleosome anchors and reservoirs for long terminal repeat (LTR) retrotransposons, structurally resembling *Schizosaccharomyces pombe* and *C. albicans* rather than the point centromeres of *S. cerevisiae* [15,81]. Centromeric analysis reveals a karyotype dominated by acrocentric and submetacentric chromosomes, with the exception of the telocentric Chromosome V [15]. Within this framework, the *MAT* locus architecture is significantly reorganized. Breaking from the conserved synteny of standard budding yeasts [14,82], *S. schoenii* shows decoupling of the *DIC1* gene and the distribution of *SLA2-MAT* cassettes across all six chromosomes, forming a 43 kb footprint [15]. These cassettes reside primarily in pericentromeric or subtelomeric domains. In these usually repressed regions, co-transcription with the *SLA2* anchor gene allows them to bypass heterochromatic silencing [83], while the physical location limits recombination to ensure meiotic stability. Chromosome IV represents an exception, containing an active *MAT* locus within a macro-palindrome that likely facilitates transcriptional switching via intrachromosomal homologous recombination [15]. This configuration suggests a specialized adaptation that allows the yeast to remain self-fertile without sacrificing genomic stability.

## 5. Biocontrol Applications

As the search for natural and sustainable alternatives to chemical antifungals accelerates, *S. schoenii* and related yeasts could offer a solution. Because its predatory attack relies on physical and broad-spectrum enzymatic methods, it is significantly less susceptible to traditional drug resistance observed with single-target chemical antifungals, making it a promising biocontrol agent for both medicine and agriculture [3,4].

### 5.1. Pathogen Control in Medicine and Agriculture

*C. auris* is an emerging fungal pathogen that colonizes human skin and hospital surfaces, frequently exhibiting multi-drug resistance across the major antifungal classes, including azoles, polyenes, and echinocandins [1]. Pathogens like *C. auris* evade these drugs by upregulating efflux pumps or acquiring target-site point mutations. Because *S. schoenii* relies on direct physical penetration and rapid enzymatic degradation, the predator bypasses these traditional molecular evasion tactics [1,3]. In vitro co-cultivation assays using multi-drug-resistant clinical isolates of *C. auris* have successfully demonstrated this, revealing that *S. schoenii* rapidly attacks and kills the pathogen [3]. These in vitro results serve as a conceptual starting point. To go beyond a proof-of-concept, pre-clinical models of infection will be required. In parallel, future therapeutic strategies can benefit from isolating specific prey enzymes, e.g., SAPs and CAZymes for the development of antifungal formulations.

Agricultural use represents another potential application for biocontrol fungi. Synthetic chemical fungicides, while effective, have raised concerns due to environmental toxicity, residue accumulation on edible crops, and the emergence of fungicide-resistant mold strains [2,5]. *S. schoenii* has demonstrated efficacy against phytopathogenic molds of the genus *Penicillium*, specifically *P. italicum*, *P. digitatum*, and *P. expansum*, which are the primary causal agents of devastating post-harvest decay in commercial citrus fruits and apples. When applied directly to the surface of mature oranges that had been artificially inoculated with highly virulent *Penicillium* spores, *S. schoenii* successfully survived, colonized, and proliferated on the fruit surface for up to 21 days [4]. During this period, it achieved continuous pathogen suppression, significantly reducing the incidence and severity of the mold [4]. This biological control was achieved without causing any necrotic lesions or detrimental physiological changes to the plant tissue itself. Because *S. schoenii* targets specific fungal cell wall components, such as chitin and glucans, that are absent in plant cell walls, it acts as a safe, highly specific treatment. Moreover, other members of the *Saccharomycopsis* genus have been used safely in food production (such as *S. fibuligera* in rice wine fermentation) [14,61].

### 5.2. Commercial Formulation and Regulatory Challenges

Translating *S. schoenii* from the controlled laboratory environment into a commercially viable product requires overcoming significant development, formulation and regulatory challenges. For agricultural use, yeast cells applied to leaf or fruit surfaces face severe abiotic stresses, including desiccation, osmotic shock, and intense ultraviolet (UV) radiation [84]. Commercial viability depends on preservation techniques like lyophilization or spray-drying, which induce massive cellular stress. To overcome this, bioprocessing strategies can exploit the yeast’s intrinsic molecular defenses. For example, sub-lethal pre-conditioning can force the accumulation of osmo-protectants, such as disaccharide trehalose, which acts as a chemical chaperone to prevent membrane collapse during severe desiccation [85]. Commercial formulations may also require UV-blocking agents and surfactants to ensure shelf life and proper adhesion to crop cuticles [84]. Genome engineering and in-lab evolution may be useful to optimize these phenotypes [86,87].

Beyond physical formulation, securing approval from regulatory bodies like the Environmental Protection Agency (EPA) and the European Food Safety Authority (EFSA) requires rigorous risk assessment [88,89]. Although the genome lacks known mycotoxin clusters, metabolomic profiling and cytotoxicity assays are needed to definitively rule out the production of novel toxins before commercial deployment [9]. Another unique regulatory hurdle for *S. schoenii* involves its taxonomic lineage. As a member of the CUG-Ser2 clade, it shares a distant evolutionary relationship with the CUG-Ser1 clade, which contains multi-drug-resistant human pathogens such as *C. albicans* and *C. auris* [14]. Therefore, proving the strain is completely safe would be a strict regulatory requirement. The innate biological constraints of *S. schoenii*, specifically its lack of thermal tolerance for mammalian body temperatures and its obligate methionine auxotrophy [9,13], serve as natural biocontainment mechanisms that prevent unchecked environmental or clinical spread. Nevertheless, the appropriate preclinical and clinical safety studies will be required.

### 5.3. Biosafety Profile

Predatory yeasts possess several innate biological characteristics that act as robust natural biocontainment mechanisms. First, human pathogenesis typically requires thermal tolerance, specifically the ability to undergo robust vegetative growth and morphological switching at the human body temperature of 37 °C [90]. Many environmental yeast isolates fail to proliferate at this elevated temperature, severely limiting their potential as opportunistic clinical pathogens. Second, the methionine auxotrophy in *S. schoenii* serves as an ecological constraint [9,13]. The yeast also cannot proliferate uncontrollably in environments lacking complex, readily available organosulfur sources and this metabolic dependency ensures that once the immediate fungal prey population is consumed and the local organic sulfur pool is depleted, the predator population will naturally collapse, preventing unchecked environmental spread.

*S. schoenii* offers a distinct biosafety advantage over traditional filamentous biocontrol agents like *Trichoderma*. *Trichoderma* species secrete diverse secondary metabolites, including peptaibols and harzianic acid [7,8]. While effective against target molds, these bioactive compounds can occasionally exhibit broad-spectrum cytotoxicity or unintended off-target effects. In contrast, *S. schoenii* primarily uses a highly localized mechanical and enzymatic method (relying on actin-driven penetration pegs and secreted aspartic proteases) to breach its prey [9,16]. This reliance on physical, contact-dependent predation rather than chemical warfare significantly streamlines the toxicological risk assessment, making predatory yeasts highly attractive candidates for the next generation of safe, sustainable biocontrol technologies.

## 6. Conclusions

Necrotrophic mycoparasitism in budding yeasts serves as an excellent, manipulatable model for understanding the molecular mechanisms underlying microbial predatory behavior [9]. Evolutionarily locked into a predatory lifestyle owing to the complete loss of the canonical sulfate assimilation pathway, *Saccharomycopsis schoenii* relies on an expanded genomic toolkit of secreted aspartic proteases, structurally unique regional centromeres, and rewired MAPK signaling networks to hunt, penetrate, and digest its hosts [9,15,16]. The capacity of *S. schoenii* to bypass traditional drug-resistance mechanisms through physical predation positions it as a promising biological control agent for managing clinical superbugs like *Candida auris* and reducing agricultural postharvest diseases caused by *Penicillium* species [3,4].

Despite its potential, critical knowledge gaps remain. The precise molecular mechanisms and surface receptors *S. schoenii* uses for prey recognition are currently unknown [9]. Additionally, while *S. schoenii* relies on a transcriptomic burst of SAPs during an attack [9], significant biochemical questions remain, such as whether the yeast tailors this enzymatic cocktail based on the specific prey species it encounters. Targeted functional assays are needed to determine if these paralogs have specialized enzymatic roles [15]. Finally, long-term ecological studies must validate its environmental persistence, host specificity, and non-target effects within complex natural microbiomes [88]. By expanding these genomic foundations using emerging transcriptomics, CRISPR-based genome editing, and functional fitness profiling, the scientific community can accelerate the data-driven design of targeted, eco-friendly biocontrol strategies capable of addressing the increasing global crises of multi-drug resistant fungal pathogens.

## Figures and Tables

**Table 1:** Functional, morphological, and molecular distinctions between biotrophs and necrotrophs.

Feature	Biotrophism	Necrotrophism
**Trophic nature and host status**	• Derives sustained nutrition from live host cells; • Maintains host viability to retain nutrient sink long enough to absorb nutrients and reproduce intracellularly (e.g., *Ampelomyces* spp. typically induces complete atrophy of host powdery mildew colonies within 5–10 days) [40,41,42].	• Derives nutrition by killing host cells; • Rapidly destroys tissue to access cellular contents [40].
**Infection and feeding structures**	Specialized structures required for interacting with the host: • **Haustoria:** Pathogen folds inward into the host cell to create a feeding structure (surrounded by a host-derived membrane) for continuous nutrient pumping (e.g., in rusts and mildews) [40]; • **Intracellular infection hyphae:** Specialized hyphae that grow through living cells without killing them immediately (e.g., in smuts like *Ustilago maydis*) [41]; • **Arbuscule-like structures:** Highly branched feeding structures used for nutrient absorption (morphologically similar to mutualistic mycorrhizae) [41]; • **Intercellular hyphae:** Grows through microscopic gaps between plant cells (apoplast) without causing tissue collapse [41]; • **Fusion/contact sites:** Simple hyphal contact or minor penetration also occurs without specialized structures, to siphon nutrients [42,43,44]. Note: Appressoria are also widely utilized by biotrophic fungi (e.g., rusts and powdery mildews) to achieve initial surface attachment and facilitate subsequent haustorial entry [45].	Lacks specialized internal feeding structures like haustoria and instead relies on specialized infection/penetration structures alongside enzymatic force to penetrate, kill, and absorb nutrients through hyphae: • **Appressoria:** Flat, high-pressure infection structures used to mechanically breach the host cuticle/cell wall [40]; • **Penetration pegs:** Narrow hyphal projections that puncture the cell wall beneath the appressorium [3,46]; • **Invasive/vegetative hyphae:** Conventional hyphae that rapidly enter through dead tissue to absorb free nutrients [40]; • **Mycoparasitic-specific structures:** Necrotrophic mycoparasites use hook-like structures, papilla-like outgrowths, and hyphal coiling (wrapping tightly around the host fungus) to physically crush and penetrate prey [47].
**Molecular tools and secretions**	• **Effectors:** Small secreted proteins that enter the host cell to suppress immune signaling and halt programmed cell death, allowing the pathogen to establish a stable, nutrient source within the host tissue (e.g., *Ustilago maydis* uses Pep1 to block the plant’s oxidative burst and prevent cell death) [29,48]; • **Minimal CWDEs:** Highly restricted secretion of CWDEs to allow localized entry without triggering massive damage sensors [28].	• **CWDE secretion:** Chitinases, pectinases, cellulases, and xylanases damage host structures [40,47]; • **Host-specific toxins (HSTs):** Toxins that target specific susceptibility genes in host cultivars (e.g., Victorin, ToxA) [49]; • **Non-specific toxins:** Broadly toxic secondary metabolites (e.g., botrydial, non-ribosomal peptides) [49]; • **ROS:** Actively generates an oxidative burst to accelerate host cell and tissue death [49].
**Host defense and HR interaction**	• **Defense and programmed cell death:** Host localized programmed cell death (hypersensitive response or HR) is highly effective against biotrophs. By committing localized cell suicide, the host starves the pathogen [40,50].	• **Defense and programmed cell death:** Host triggers broad-spectrum stress responses. Necrotrophs can actively hijack these host pathways to induce localized cell death, rapidly generating a pool of dead cellular material for nutrient absorption [40,51].
**Host range and specificity**	**Specific (specialists):** Usually restricted to a single host species (or even a specific genetic cultivar) due to complex “gene-for-gene” recognition requirements [41,52].	**Broad (generalists):** Often capable of infecting a wide variety of plant or fungal families [40] (though some exceptions exist for necrotrophs that rely on highly specific HSTs [49]).
**In vitro cultivation**	Generally impossible to culture on artificial media in a laboratory without the living host [40].	Readily cultured in a laboratory on standard media [40,46]. This capacity classifies many necrotrophs as facultative parasites able to shift to saprotrophic growth in the absence of prey.
**Literature examples (plant pathogens)**	• *Blumeria graminis* (Powdery mildew); • *Puccinia graminis* (Stem rust of wheat); • *Plasmopara viticola* (Downy mildew—an Oomycete); • *Melampsora lini* (Flax rust) [40,41].	• *Botrytis cinerea* (Gray mold); • *Sclerotinia sclerotiorum* (White mold); • *Alternaria brassicicola* (Dark leaf spot); • *Cochliobolus heterostrophus* (Southern corn leaf blight) [40,49].
**Literature examples (mycoparasites)**	• *Ampelomyces quisqualis* (anamorphic fungus that is a hyperparasite of powdery mildews—without rapidly killing them) [42,44]; • *Sphaerodes quadrangularis* (Facultative biotroph of *Fusarium* species) [53].	• *Trichoderma* spp. (e.g., *Trichoderma harzianum*, *T. atroviride*—aggressively coil, lyse, and consume prey fungi) [47]; • *Clonostachys rosea* (Uses hook structures to penetrate and disintegrate *B. cinerea*) [38].

## Data Availability

No new data were created or analyzed in this study. Data sharing is not applicable to this article.

## Abbreviations

The following abbreviations are used in this manuscript:

CAZymes	Carbohydrate-active enzymes
CWDE	Cell wall-degrading enzyme
EFSA	European food safety authority
EPA	Environmental protection agency
GH	Glycoside hydrolase
Hi-C	High-throughput chromosome conformation capture
HR	Hypersensitive response
HST	Host-specific toxin
LTR	Long terminal repeat
MAPK	Mitogen-activated protein kinase
MAT	Mating-type
Mb	Megabase
NLP	Necrosis and ethylene-inducing protein
ROS	Reactive oxygen species
SAP	Secreted aspartic proteases
SSP	Small secreted protein
UV	Ultraviolet
